# Guanidinium 2-(myristoylsulfanyl)ethane­sulfonate

**DOI:** 10.1107/S1600536811046472

**Published:** 2011-11-12

**Authors:** Elizabeth S. Monillas, Wesley H. Monillas, Eric R. Sirianni, Glenn P. A. Yap, Klaus H. Theopold

**Affiliations:** a Department of Chemistry and Biochemistry, University of Delaware, Newark, DE 19716, USA

## Abstract

In the title compound, CH_6_N_3_^+^·C_16_H_31_O_4_S_2_^−^ [systematic name: guanidinium 2-(tetra­deca­noylsulfan­yl)ethane­sulfon­ate], each 2-(myristoyl­thio)­ethane­sulfonate ion displays hydrogen bonding to three guanidinium counter-ions, which themselves display hydrogen bonding to two symmetry-related 2-(myristoylthio)ethanesulfonate ions. Thus each cation forms six N—H⋯O bonds to neighboring anions, thereby self-assembling an extended ladder-type network. The average hydrogen-bond donor–acceptor distance is 2.931 (5) Å. The alkyl chains form the rungs of a ladder with hydrogen-bonding inter­actions forming the side rails.

## Related literature

The synthesis of the title compound was adapted from Schramm *et al.* (1954 ▶) and Dalton *et al.* (1981 ▶). For extended networks *via* hydrogen-bonding in guanidinium organo­sulfonates, see: Horner *et al.* (2001 ▶, 2007 ▶); Russell & Ward (1996 ▶). For typical donor-acceptor distances in these compounds, see: Adams (1978 ▶); Ashiq *et al.* (2010 ▶). For studies of these structural motifs for use as electronic materials, see: Russell *et al.* (1994 ▶). 
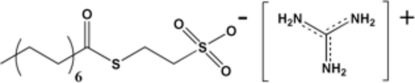


## Experimental

### 

#### Crystal data

CH_6_N_3_^+^·C_16_H_31_O_4_S_2_^−^
*M* *_r_* = 411.62
Monoclinic, 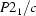

*a* = 25.185 (13) Å
*b* = 7.370 (4) Å
*c* = 12.663 (7) Å
β = 101.851 (10)°
*V* = 2300 (2) Å^3^
*Z* = 4
Mo *K*α radiation
μ = 0.26 mm^−1^
*T* = 200 K
0.25 × 0.18 × 0.01 mm

#### Data collection

Bruker APEX diffractometer
Absorption correction: multi-scan (*SADABS*; Sheldrick, 2003 ▶) *T*_min_ = 0.938, *T*_max_ = 0.997
18707 measured reflections
5688 independent reflections
2763 reflections with *I* > 2σ(*I*)
*R*_int_ = 0.085

#### Refinement

*R*[*F* ^2^ > 2σ(*F* ^2^)] = 0.082
*wR*(*F* ^2^) = 0.247
*S* = 1.01
5688 reflections
292 parameters
83 restraints
H atoms treated by a mixture of independent and constrained refinement
Δρ_max_ = 0.45 e Å^−3^
Δρ_min_ = −0.39 e Å^−3^

### 

Data collection: *SMART* (Bruker, 2003 ▶); cell refinement: *SAINT* (Bruker, 2003 ▶); data reduction: *SAINT*; program(s) used to solve structure: *SHELXS97* (Sheldrick, 2008 ▶); program(s) used to refine structure: *SHELXL97* (Sheldrick, 2008 ▶); molecular graphics: *SHELXTL* (Sheldrick, 2008 ▶); software used to prepare material for publication: *SHELXTL*.

## Supplementary Material

Crystal structure: contains datablock(s) I, global. DOI: 10.1107/S1600536811046472/zj2033sup1.cif

Structure factors: contains datablock(s) I. DOI: 10.1107/S1600536811046472/zj2033Isup3.hkl

Supplementary material file. DOI: 10.1107/S1600536811046472/zj2033Isup3.cml

Additional supplementary materials:  crystallographic information; 3D view; checkCIF report

## Figures and Tables

**Table 1:** Hydrogen-bond geometry (Å, °)

*D*—H⋯*A*	*D*—H	H⋯*A*	*D*⋯*A*	*D*—H⋯*A*
N1—H1⋯O1^i^	0.87 (4)	2.12 (4)	2.943 (4)	159 (4)
N1—H2⋯O3^ii^	0.80 (4)	2.11 (4)	2.900 (4)	171 (4)
N2—H3⋯O2^ii^	0.84 (4)	2.12 (4)	2.957 (4)	171 (4)
N2—H4⋯O1	0.84 (4)	2.13 (4)	2.960 (4)	172 (4)
N3—H5⋯O2	0.83 (5)	2.06 (5)	2.892 (4)	178 (5)
N3—H6⋯O3^i^	0.82 (5)	2.14 (5)	2.942 (4)	167 (5)

Symmetry codes: (i) 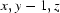; (ii) 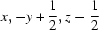.

**Fig. 1.**
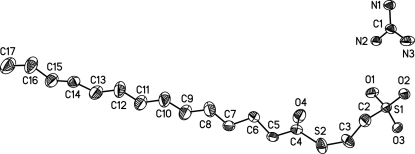
Molecular diagram of the structure in the asymmetric unit omitting H-atoms and one of two disordered contributions for clarity. Thermal ellipsoids depicted at 50% probability.

**Fig. 2.**
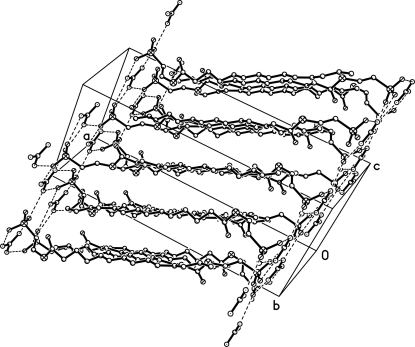
Packing diagram displaying extended ladder network.

## supplementary crystallographic information

### Comment

The title compound was prepared as a reagent in attempts to synthesized a myristoylate protein derivative *in vitro*. The synthesis was adapted from Schramm *et al.* (1954) and Dalton *et al.* (1981). During characterization by X-ray diffraction it was observed to display an interesting ladder-type lattice network. It has been previously reported that guanidinium organosulfonates are capable of extended networks *via* hydrogen-bonding (Russell & Ward 1996, Horner *et al.* 2001, 2007). As shown in Figure 2 the guanidinium counterions form near planar end caps, the side rails, with the inward facing myristoyl groups interlocking causing a bilayer stacking or the rungs of the extended ladder-type network. The average hydrogen bond donor-acceptor distance is 2.931 (5) Å which is in the typical range observed for these type of compounds (Adams 1978, Ashiq *et al.* 2010). These structural motifs have previously been studied for use as electronic materials (Russell *et al.* 1994).

### Experimental

The compound synthesis was adapted from Schramm *et al.* (1954) and Dalton *et al.* (1981). Guanidinium 2-mercaptoethansulfonate, 1.0 g (5 mmol), and guanidinium carbonate, 0.9 g (9.9 mmol), were added to 20 mL 1:1 acetonitrile/water. The mixture was stirred and purged with dry nitrogen gas. When the guanidinium carbonate completely dissolved, 1.36 mL (5.01 mmol) of myristoyl chloride was added and the reaction was stirred under 1 atmosphere of nitrogen. After one hour,  4 mL 1:1 acetonitrile/water were added to the mixture. The mixture was stirred for an additional hour after which time the guanidinium 2-(myristoylthio)ethanesulfonate precipitate was filtered and collected yielding 1.09 g (2.65 mmol, 53% yield) of product. Crystals suitable for X-ray diffraction were obtained from slow evaporation of a saturated solution of the compound from a 9:1 acetonitrile/water mixed solvent.

### Refinement

All non-hydrogen atoms were refined with anisotropic displacement parameters. Hydrogen atoms on the guanidium ion were located and refined with 1.2 *U_eq_* of the attached N atom. All other H-atoms were were placed in calculated positions Chemically identical atoms in the disordered portions of the anion constrained to similar 1,2 and 1,3 atom-atom separations, equal atomic displacement parameters, rigid bond restraints and refined to roughly 50/50 site occupancy ratio. Although several C level alerts occur in the checkCIF report, trial refinements with global rigid bond constraints did not significantly improve the structure.

### Crystal data

**Table:**

CH_6_N_3_^+^·C_16_H_31_O_4_S_2_^−^	*F*(000) = 896
*M**_r_* = 411.62	*D*_x_ = 1.189 Mg m^−^^3^
Monoclinic, *P*2_1_/*c*	Melting point: 326 K
Hall symbol: -P 2ybc	Mo *K*α radiation, λ = 0.71073 Å
*a* = 25.185 (13) Å	Cell parameters from 4043 reflections
*b* = 7.370 (4) Å	θ = 1.7–25.0°
*c* = 12.663 (7) Å	µ = 0.26 mm^−^^1^
β = 101.851 (10)°	*T* = 200 K
*V* = 2300 (2) Å^3^	Block, colourless
*Z* = 4	0.25 × 0.18 × 0.01 mm

### Data collection

**Table:**

Bruker APEX diffractometer	5688 independent reflections
Radiation source: fine-focus sealed tube	2763 reflections with *I* > 2σ(*I*)
graphite	*R*_int_ = 0.085
Detector resolution: 836.6 pixels mm^-1^	θ_max_ = 28.4°, θ_min_ = 1.7°
ω scans	*h* = −33→31
Absorption correction: multi-scan (*SADABS*; Sheldrick, 2003)	*k* = −9→9
*T*_min_ = 0.938, *T*_max_ = 0.997	*l* = −16→16
18707 measured reflections	

### Refinement

**Table:**

Refinement on *F*^2^	Primary atom site location: structure-invariant direct methods
Least-squares matrix: full	Secondary atom site location: difference Fourier map
*R*[*F*^2^ > 2σ(*F*^2^)] = 0.082	Hydrogen site location: inferred from neighbouring sites
*wR*(*F*^2^) = 0.247	H atoms treated by a mixture of independent and constrained refinement
*S* = 1.01	*w* = 1/[σ^2^(*F*_o_^2^) + (0.1*P*)^2^ + 2.2088*P*] where *P* = (*F*_o_^2^ + 2*F*_c_^2^)/3
5688 reflections	(Δ/σ)_max_ < 0.001
292 parameters	Δρ_max_ = 0.45 e Å^−^^3^
83 restraints	Δρ_min_ = −0.39 e Å^−^^3^

### Special details

**Table:**

Experimental. Data collection is performed with four batch runs at φ = 0.00 ° (600 frames), at φ = 90.00 ° (600 frames), at φ = 180 ° (600 frames) and at φ = 270 ° (600 frames). Frame width = 0.30 \& in ω. Data is merged, corrected for decay, and treated with multi-scan absorption corrections.
Geometry. All e.s.d.'s (except the e.s.d. in the dihedral angle between two l.s. planes) are estimated using the full covariance matrix. The cell e.s.d.'s are taken into account individually in the estimation of e.s.d.'s in distances, angles and torsion angles; correlations between e.s.d.'s in cell parameters are only used when they are defined by crystal symmetry. An approximate (isotropic) treatment of cell e.s.d.'s is used for estimating e.s.d.'s involving l.s. planes.
Refinement. Refinement of *F*^2^ against ALL reflections. The weighted *R*-factor *wR* and goodness of fit *S* are based on *F*^2^, conventional *R*-factors *R* are based on *F*, with *F* set to zero for negative *F*^2^. The threshold expression of *F*^2^ > σ(*F*^2^) is used only for calculating *R*-factors(gt) *etc*. and is not relevant to the choice of reflections for refinement. *R*-factors based on *F*^2^ are statistically about twice as large as those based on *F*, and *R*- factors based on ALL data will be even larger.

### Fractional atomic coordinates and isotropic or equivalent isotropic displacement parameters (Å^2^)

**Table:**

	*x*	*y*	*z*	*U*_iso_*/*U*_eq_	Occ. (<1)
S1	0.08990 (4)	0.52011 (11)	0.43991 (7)	0.0377 (3)	
N1	0.05207 (15)	−0.1418 (4)	0.2036 (3)	0.0416 (8)	
H1	0.0523 (16)	−0.240 (6)	0.242 (3)	0.050*	
H2	0.0514 (17)	−0.145 (6)	0.140 (3)	0.050*	
N2	0.05787 (14)	0.1693 (4)	0.2060 (3)	0.0391 (8)	
H3	0.0609 (16)	0.173 (5)	0.141 (3)	0.047*	
H4	0.0627 (16)	0.264 (6)	0.243 (3)	0.047*	
N3	0.05382 (19)	0.0153 (5)	0.3608 (3)	0.0571 (11)	
H5	0.0607 (19)	0.111 (7)	0.395 (4)	0.069*	
H6	0.0504 (19)	−0.080 (7)	0.392 (4)	0.069*	
O1	0.08125 (13)	0.5176 (3)	0.3228 (2)	0.0514 (8)	
O2	0.07864 (11)	0.3453 (3)	0.48467 (19)	0.0428 (7)	
O3	0.06170 (10)	0.6702 (3)	0.47983 (19)	0.0399 (7)	
C1	0.05490 (16)	0.0131 (5)	0.2569 (3)	0.0362 (8)	
C2	0.15997 (19)	0.5593 (6)	0.4892 (5)	0.0568 (12)	
H2A	0.1790 (17)	0.452 (6)	0.460 (3)	0.056 (12)*	
H2B	0.163 (2)	0.543 (6)	0.561 (4)	0.071 (16)*	
C4	0.2617 (4)	0.7903 (14)	0.3451 (6)	0.0384 (15)	0.4984 (16)
C5	0.3212 (4)	0.8295 (14)	0.3533 (7)	0.0417 (17)	0.4984 (16)
H5A	0.3416	0.7860	0.4241	0.050*	0.4984 (16)
H5B	0.3264	0.9625	0.3507	0.050*	0.4984 (16)
C6	0.3447 (4)	0.7415 (18)	0.2645 (7)	0.0376 (18)	0.4984 (16)
H6A	0.3218	0.7726	0.1935	0.045*	0.4984 (16)
H6B	0.3442	0.6080	0.2727	0.045*	0.4984 (16)
C7	0.4024 (4)	0.804 (2)	0.2678 (8)	0.043 (2)	0.4984 (16)
H7A	0.4009	0.9353	0.2512	0.052*	0.4984 (16)
H7B	0.4226	0.7906	0.3432	0.052*	0.4984 (16)
C24	0.2725 (4)	0.7552 (13)	0.3933 (6)	0.0384 (15)	0.5016 (16)
C25	0.3012 (4)	0.8162 (14)	0.3060 (7)	0.0417 (17)	0.5016 (16)
H25A	0.3063	0.9493	0.3117	0.050*	0.5016 (16)
H25B	0.2775	0.7904	0.2350	0.050*	0.5016 (16)
C26	0.3560 (4)	0.7276 (17)	0.3097 (7)	0.0376 (18)	0.5016 (16)
H26A	0.3509	0.5950	0.2998	0.045*	0.5016 (16)
H26B	0.3794	0.7484	0.3816	0.045*	0.5016 (16)
C27	0.3847 (5)	0.801 (2)	0.2231 (7)	0.043 (2)	0.5016 (16)
H27A	0.3576	0.7993	0.1543	0.052*	0.5016 (16)
H27B	0.3930	0.9299	0.2408	0.052*	0.5016 (16)
C8	0.4335 (2)	0.7213 (6)	0.2010 (5)	0.0800 (17)	
H8A	0.4066	0.6723	0.1395	0.096*	
H8B	0.4501	0.6143	0.2423	0.096*	
C9	0.4748 (2)	0.7936 (6)	0.1544 (5)	0.0821 (18)	
H9A	0.4554	0.8766	0.0980	0.098*	
H9B	0.4953	0.8733	0.2115	0.098*	
C10	0.5146 (2)	0.7172 (7)	0.1078 (5)	0.088 (2)	
H10A	0.4942	0.6379	0.0505	0.106*	
H10B	0.5340	0.6338	0.1640	0.106*	
C11	0.5558 (2)	0.7898 (6)	0.0618 (5)	0.0740 (16)	
H11A	0.5366	0.8746	0.0063	0.089*	
H11B	0.5767	0.8675	0.1195	0.089*	
C12	0.5952 (2)	0.7120 (7)	0.0142 (6)	0.098 (2)	
H12A	0.5741	0.6355	−0.0439	0.117*	
H12B	0.6138	0.6258	0.0695	0.117*	
C13	0.6371 (2)	0.7811 (6)	−0.0313 (4)	0.0693 (15)	
H13A	0.6507	0.8881	0.0134	0.083*	
H13B	0.6187	0.8300	−0.1023	0.083*	
C14	0.6846 (4)	0.6936 (15)	−0.0512 (7)	0.0436 (17)	0.4984 (16)
H14A	0.6734	0.5684	−0.0738	0.052*	0.4984 (16)
H14B	0.7095	0.6830	0.0200	0.052*	0.4984 (16)
C34	0.6675 (3)	0.6978 (15)	−0.1047 (7)	0.0436 (17)	0.5016 (16)
H34A	0.6730	0.5698	−0.0812	0.052*	0.5016 (16)
H34B	0.6424	0.6949	−0.1760	0.052*	0.5016 (16)
C15	0.7177 (2)	0.7554 (6)	−0.1253 (5)	0.0736 (15)	
H15A	0.6916	0.8067	−0.1873	0.088*	
H15B	0.7382	0.8603	−0.0887	0.088*	
C16	0.7555 (3)	0.6604 (7)	−0.1720 (6)	0.104 (2)	
H16A	0.7742	0.5806	−0.1131	0.125*	
H16B	0.7327	0.5784	−0.2245	0.125*	
C17	0.7963 (3)	0.7127 (8)	−0.2238 (6)	0.111 (2)	
H17A	0.8139	0.6046	−0.2462	0.166*	
H17B	0.8232	0.7851	−0.1745	0.166*	
H17C	0.7809	0.7854	−0.2875	0.166*	
S2	0.24330 (9)	0.7885 (4)	0.47364 (18)	0.0565 (5)	0.4984 (16)
O4	0.2287 (2)	0.7707 (10)	0.2631 (4)	0.0637 (18)	0.4984 (16)
C3	0.1879 (3)	0.7406 (17)	0.4910 (9)	0.0534 (19)	0.4984 (16)
H3A	0.1865	0.7925	0.5625	0.064*	0.4984 (16)
H3B	0.1628	0.8153	0.4378	0.064*	0.4984 (16)
S22	0.20209 (9)	0.7959 (4)	0.36378 (18)	0.0565 (5)	0.5016 (16)
O24	0.2953 (2)	0.6859 (9)	0.4769 (4)	0.0584 (16)	0.5016 (16)
C23	0.1698 (4)	0.7495 (17)	0.4390 (8)	0.0534 (19)	0.5016 (16)
H23A	0.1330	0.7943	0.4062	0.064*	0.5016 (16)
H23B	0.1830	0.8289	0.5019	0.064*	0.5016 (16)

### Atomic displacement parameters (Å^2^)

**Table:**

	*U*^11^	*U*^22^	*U*^33^	*U*^12^	*U*^13^	*U*^23^
S1	0.0548 (6)	0.0278 (5)	0.0337 (5)	−0.0039 (4)	0.0167 (4)	−0.0003 (4)
N1	0.066 (2)	0.0291 (16)	0.0317 (17)	−0.0027 (16)	0.0143 (17)	−0.0002 (14)
N2	0.056 (2)	0.0286 (17)	0.0352 (18)	−0.0036 (15)	0.0150 (16)	−0.0022 (13)
N3	0.114 (4)	0.0287 (17)	0.0329 (18)	−0.005 (2)	0.024 (2)	−0.0012 (14)
O1	0.095 (2)	0.0311 (14)	0.0330 (14)	−0.0013 (14)	0.0238 (14)	0.0015 (11)
O2	0.0652 (18)	0.0293 (13)	0.0364 (14)	−0.0084 (13)	0.0166 (13)	0.0037 (10)
O3	0.0531 (17)	0.0329 (13)	0.0379 (14)	0.0008 (12)	0.0188 (12)	−0.0006 (10)
C1	0.046 (2)	0.0285 (18)	0.0342 (18)	−0.0014 (16)	0.0083 (16)	0.0005 (15)
C2	0.052 (3)	0.049 (3)	0.077 (4)	0.000 (2)	0.030 (3)	−0.008 (2)
C4	0.042 (4)	0.035 (4)	0.036 (4)	−0.003 (3)	0.002 (3)	−0.004 (4)
C5	0.051 (6)	0.035 (3)	0.040 (5)	−0.010 (4)	0.010 (4)	−0.008 (4)
C6	0.039 (4)	0.036 (3)	0.036 (6)	−0.002 (3)	0.004 (4)	0.002 (5)
C7	0.054 (7)	0.038 (2)	0.039 (6)	−0.008 (5)	0.011 (4)	−0.005 (6)
C24	0.042 (4)	0.035 (4)	0.036 (4)	−0.003 (3)	0.002 (3)	−0.004 (4)
C25	0.051 (6)	0.035 (3)	0.040 (5)	−0.010 (4)	0.010 (4)	−0.008 (4)
C26	0.039 (4)	0.036 (3)	0.036 (6)	−0.002 (3)	0.004 (4)	0.002 (5)
C27	0.054 (7)	0.038 (2)	0.039 (6)	−0.008 (5)	0.011 (4)	−0.005 (6)
C8	0.064 (3)	0.040 (2)	0.152 (5)	−0.001 (2)	0.057 (4)	−0.006 (3)
C9	0.106 (4)	0.035 (2)	0.129 (5)	−0.003 (3)	0.081 (4)	0.002 (3)
C10	0.079 (4)	0.041 (3)	0.167 (6)	−0.003 (3)	0.077 (4)	−0.011 (3)
C11	0.091 (4)	0.039 (2)	0.111 (4)	0.000 (2)	0.065 (3)	0.006 (3)
C12	0.089 (4)	0.039 (3)	0.193 (7)	−0.004 (3)	0.095 (5)	−0.008 (3)
C13	0.091 (4)	0.040 (2)	0.093 (4)	−0.001 (2)	0.055 (3)	0.001 (2)
C14	0.051 (5)	0.036 (2)	0.044 (5)	0.003 (4)	0.011 (4)	0.002 (5)
C34	0.051 (5)	0.036 (2)	0.044 (5)	0.003 (4)	0.011 (4)	0.002 (5)
C15	0.085 (4)	0.046 (3)	0.108 (4)	−0.005 (3)	0.059 (3)	−0.005 (3)
C16	0.101 (5)	0.045 (3)	0.195 (7)	−0.001 (3)	0.100 (5)	−0.003 (4)
C17	0.125 (6)	0.074 (4)	0.165 (7)	−0.012 (4)	0.102 (5)	−0.019 (4)
S2	0.0356 (8)	0.0827 (12)	0.0492 (9)	−0.0116 (8)	0.0037 (7)	0.0106 (8)
O4	0.042 (3)	0.103 (5)	0.044 (3)	0.001 (3)	0.003 (3)	−0.017 (3)
C3	0.024 (4)	0.056 (3)	0.075 (6)	−0.007 (4)	−0.003 (4)	−0.002 (5)
S22	0.0356 (8)	0.0827 (12)	0.0492 (9)	−0.0116 (8)	0.0037 (7)	0.0106 (8)
O24	0.053 (4)	0.075 (4)	0.043 (3)	0.005 (3)	0.001 (3)	0.015 (3)
C23	0.024 (4)	0.056 (3)	0.075 (6)	−0.007 (4)	−0.003 (4)	−0.002 (5)

### Geometric parameters (Å, °)

**Table:**

S1—O1	1.454 (3)	C8—C9	1.402 (6)
S1—O2	1.459 (2)	C8—H8A	0.9900
S1—O3	1.459 (3)	C8—H8B	0.9900
S1—C2	1.771 (5)	C9—C10	1.382 (6)
N1—C1	1.321 (5)	C9—H9A	0.9900
N1—H1	0.87 (4)	C9—H9B	0.9900
N1—H2	0.80 (4)	C10—C11	1.397 (6)
N2—C1	1.329 (4)	C10—H10A	0.9900
N2—H3	0.84 (4)	C10—H10B	0.9900
N2—H4	0.84 (4)	C11—C12	1.385 (6)
N3—C1	1.322 (5)	C11—H11A	0.9900
N3—H5	0.83 (5)	C11—H11B	0.9900
N3—H6	0.82 (5)	C12—C13	1.398 (6)
C2—C3	1.507 (12)	C12—H12A	0.9900
C2—C23	1.580 (12)	C12—H12B	0.9900
C2—H2A	1.03 (4)	C13—C14	1.426 (9)
C2—H2B	0.90 (5)	C13—C34	1.457 (9)
C4—O4	1.199 (9)	C13—H13A	0.9900
C4—C5	1.508 (11)	C13—H13B	0.9900
C4—S2	1.781 (9)	C14—C15	1.452 (9)
C5—C6	1.520 (10)	C14—H14A	0.9900
C5—H5A	0.9900	C14—H14B	0.9900
C5—H5B	0.9900	C34—C15	1.409 (9)
C6—C7	1.516 (11)	C34—H34A	0.9900
C6—H6A	0.9900	C34—H34B	0.9900
C6—H6B	0.9900	C15—C16	1.405 (6)
C7—C8	1.405 (14)	C15—H15A	0.9900
C7—H7A	0.9900	C15—H15B	0.9900
C7—H7B	0.9900	C16—C17	1.384 (7)
C24—O24	1.209 (8)	C16—H16A	0.9900
C24—C25	1.507 (10)	C16—H16B	0.9900
C24—S22	1.762 (9)	C17—H17A	0.9800
C25—C26	1.520 (11)	C17—H17B	0.9800
C25—H25A	0.9900	C17—H17C	0.9800
C25—H25B	0.9900	S2—C3	1.500 (9)
C26—C27	1.529 (11)	C3—H3A	0.9900
C26—H26A	0.9900	C3—H3B	0.9900
C26—H26B	0.9900	S22—C23	1.415 (9)
C27—C8	1.440 (14)	C23—H23A	0.9900
C27—H27A	0.9900	C23—H23B	0.9900
C27—H27B	0.9900		
			
O1—S1—O2	112.64 (15)	C10—C9—H9A	103.8
O1—S1—O3	112.41 (16)	C8—C9—H9A	103.8
O2—S1—O3	112.79 (15)	C10—C9—H9B	103.8
O1—S1—C2	106.9 (2)	C8—C9—H9B	103.8
O2—S1—C2	105.5 (2)	H9A—C9—H9B	105.4
O3—S1—C2	106.0 (2)	C9—C10—C11	133.5 (5)
C1—N1—H1	116 (3)	C9—C10—H10A	103.8
C1—N1—H2	122 (3)	C11—C10—H10A	103.8
H1—N1—H2	122 (4)	C9—C10—H10B	103.8
C1—N2—H3	122 (3)	C11—C10—H10B	103.8
C1—N2—H4	118 (3)	H10A—C10—H10B	105.4
H3—N2—H4	120 (4)	C12—C11—C10	133.0 (5)
C1—N3—H5	119 (3)	C12—C11—H11A	104.0
C1—N3—H6	120 (3)	C10—C11—H11A	104.0
H5—N3—H6	120 (5)	C12—C11—H11B	104.0
N1—C1—N3	120.6 (3)	C10—C11—H11B	104.0
N1—C1—N2	120.2 (3)	H11A—C11—H11B	105.4
N3—C1—N2	119.1 (3)	C11—C12—C13	134.1 (5)
C3—C2—S1	125.3 (5)	C11—C12—H12A	103.7
C23—C2—S1	103.3 (4)	C13—C12—H12A	103.7
C3—C2—H2A	116 (2)	C11—C12—H12B	103.7
C23—C2—H2A	114 (2)	C13—C12—H12B	103.7
S1—C2—H2A	105 (2)	H12A—C12—H12B	105.3
C3—C2—H2B	99 (3)	C12—C13—C14	129.7 (6)
C23—C2—H2B	122 (3)	C12—C13—C34	130.6 (6)
S1—C2—H2B	102 (3)	C12—C13—H13A	104.8
H2A—C2—H2B	108 (4)	C14—C13—H13A	104.8
O4—C4—C5	125.8 (8)	C34—C13—H13A	122.3
O4—C4—S2	121.7 (7)	C12—C13—H13B	104.8
C5—C4—S2	112.3 (6)	C14—C13—H13B	104.8
C4—C5—C6	113.5 (8)	C34—C13—H13B	77.4
C4—C5—H5A	108.9	H13A—C13—H13B	105.8
C6—C5—H5A	108.9	C13—C14—C15	125.9 (8)
C4—C5—H5B	108.9	C13—C14—H14A	105.8
C6—C5—H5B	108.9	C15—C14—H14A	105.8
H5A—C5—H5B	107.7	C13—C14—H14B	105.8
C5—C6—C7	111.5 (9)	C15—C14—H14B	105.8
C5—C6—H6A	109.3	H14A—C14—H14B	106.2
C7—C6—H6A	109.3	C15—C34—C13	127.0 (8)
C5—C6—H6B	109.3	C15—C34—H34A	105.6
C7—C6—H6B	109.3	C13—C34—H34A	105.6
H6A—C6—H6B	108.0	C15—C34—H34B	105.6
C8—C7—C6	120.1 (9)	C13—C34—H34B	105.6
C8—C7—H7A	107.3	H34A—C34—H34B	106.1
C6—C7—H7A	107.3	C34—C15—C16	129.7 (6)
C8—C7—H7B	107.3	C16—C15—C14	130.1 (6)
C6—C7—H7B	107.3	C34—C15—H15A	77.6
H7A—C7—H7B	106.9	C16—C15—H15A	104.7
O24—C24—C25	123.7 (8)	C14—C15—H15A	104.7
O24—C24—S22	122.3 (7)	C34—C15—H15B	123.3
C25—C24—S22	114.0 (6)	C16—C15—H15B	104.7
C24—C25—C26	114.8 (8)	C14—C15—H15B	104.7
C24—C25—H25A	108.6	H15A—C15—H15B	105.7
C26—C25—H25A	108.6	C17—C16—C15	133.9 (5)
C24—C25—H25B	108.6	C17—C16—H16A	103.7
C26—C25—H25B	108.6	C15—C16—H16A	103.7
H25A—C25—H25B	107.6	C17—C16—H16B	103.7
C25—C26—C27	112.8 (9)	C15—C16—H16B	103.7
C25—C26—H26A	109.0	H16A—C16—H16B	105.4
C27—C26—H26A	109.0	C16—C17—H17A	109.5
C25—C26—H26B	109.0	C16—C17—H17B	109.5
C27—C26—H26B	109.0	H17A—C17—H17B	109.5
H26A—C26—H26B	107.8	C16—C17—H17C	109.5
C8—C27—C26	122.9 (10)	H17A—C17—H17C	109.5
C8—C27—H27A	106.6	H17B—C17—H17C	109.5
C26—C27—H27A	106.6	C3—S2—C4	124.1 (5)
C8—C27—H27B	106.6	C2—C3—S2	130.6 (8)
C26—C27—H27B	106.6	C2—C3—H3A	104.6
H27A—C27—H27B	106.6	S2—C3—H3A	104.6
C7—C8—C9	130.0 (7)	C2—C3—H3B	104.6
C7—C8—C27	26.2 (4)	S2—C3—H3B	104.6
C9—C8—C27	131.6 (7)	H3A—C3—H3B	105.7
C7—C8—H8A	104.8	C23—S22—C24	120.6 (5)
C9—C8—H8A	104.8	S22—C23—C2	130.1 (9)
C27—C8—H8A	80.1	S22—C23—H23A	104.7
C7—C8—H8B	104.8	C2—C23—H23A	104.7
C9—C8—H8B	104.8	S22—C23—H23B	104.7
C27—C8—H8B	120.4	C2—C23—H23B	104.7
H8A—C8—H8B	105.8	H23A—C23—H23B	105.7
C10—C9—C8	133.7 (5)		
			
O1—S1—C2—C3	77.5 (7)	C11—C12—C13—C14	160.2 (8)
O2—S1—C2—C3	−162.4 (6)	C11—C12—C13—C34	−161.4 (8)
O3—S1—C2—C3	−42.6 (7)	C12—C13—C14—C15	161.0 (8)
O1—S1—C2—C23	60.2 (5)	C34—C13—C14—C15	56.8 (12)
O2—S1—C2—C23	−179.7 (5)	C12—C13—C34—C15	−162.1 (8)
O3—S1—C2—C23	−59.9 (5)	C14—C13—C34—C15	−61.0 (13)
O4—C4—C5—C6	−33.4 (15)	C13—C34—C15—C16	162.0 (8)
S2—C4—C5—C6	150.1 (8)	C13—C34—C15—C14	58.6 (13)
C4—C5—C6—C7	172.8 (9)	C13—C14—C15—C34	−59.3 (13)
C5—C6—C7—C8	172.1 (9)	C13—C14—C15—C16	−161.1 (8)
O24—C24—C25—C26	20.9 (14)	C34—C15—C16—C17	157.6 (9)
S22—C24—C25—C26	−159.7 (7)	C14—C15—C16—C17	−163.9 (9)
C24—C25—C26—C27	−177.2 (9)	O4—C4—S2—C3	7.9 (12)
C25—C26—C27—C8	−171.4 (9)	C5—C4—S2—C3	−175.6 (8)
C6—C7—C8—C9	149.4 (9)	C23—C2—C3—S2	−104.3 (19)
C6—C7—C8—C27	44.5 (16)	S1—C2—C3—S2	−144.0 (7)
C26—C27—C8—C7	−58.2 (18)	C4—S2—C3—C2	74.8 (11)
C26—C27—C8—C9	−156.3 (9)	O24—C24—S22—C23	0.7 (12)
C7—C8—C9—C10	163.9 (9)	C25—C24—S22—C23	−178.6 (8)
C27—C8—C9—C10	−161.3 (8)	C24—S22—C23—C2	−59.9 (11)
C8—C9—C10—C11	−179.8 (7)	C3—C2—C23—S22	96.7 (16)
C9—C10—C11—C12	−179.1 (8)	S1—C2—C23—S22	−115.7 (8)
C10—C11—C12—C13	−179.2 (8)		

### Hydrogen-bond geometry (Å, °)

**Table:**

*D*—H···*A*	*D*—H	H···*A*	*D*···*A*	*D*—H···*A*
N1—H1···O1^i^	0.87 (4)	2.12 (4)	2.943 (4)	159 (4)
N1—H2···O3^ii^	0.80 (4)	2.11 (4)	2.900 (4)	171 (4)
N1—H2···S1^ii^	0.80 (4)	3.04 (4)	3.764 (4)	152 (4)
N2—H3···O2^ii^	0.84 (4)	2.12 (4)	2.957 (4)	171 (4)
N2—H4···O1	0.84 (4)	2.13 (4)	2.960 (4)	172 (4)
N3—H5···O2	0.83 (5)	2.06 (5)	2.892 (4)	178 (5)
N3—H6···O3^i^	0.82 (5)	2.14 (5)	2.942 (4)	167 (5)

Symmetry codes: (i) *x*, *y*−1, *z*; (ii) *x*, −*y*+1/2, *z*−1/2.
